# Local minimization of prediction errors drives learning of invariant object representations in a generative network model of visual perception

**DOI:** 10.3389/fncom.2023.1207361

**Published:** 2023-09-25

**Authors:** Matthias Brucklacher, Sander M. Bohté, Jorge F. Mejias, Cyriel M. A. Pennartz

**Affiliations:** ^1^Cognitive and Systems Neuroscience Group, Swammerdam Institute for Life Sciences, University of Amsterdam, Amsterdam, Netherlands; ^2^Machine Learning Group, Centrum Wiskunde & Informatica, Amsterdam, Netherlands

**Keywords:** self-supervised learning, predictive coding, generative model, vision, hierarchy, representation learning, Hebbian learning, video

## Abstract

The ventral visual processing hierarchy of the cortex needs to fulfill at least two key functions: perceived objects must be mapped to high-level representations invariantly of the precise viewing conditions, and a generative model must be learned that allows, for instance, to fill in occluded information guided by visual experience. Here, we show how a multilayered predictive coding network can learn to recognize objects from the bottom up and to generate specific representations via a top-down pathway through a single learning rule: the local minimization of prediction errors. Trained on sequences of continuously transformed objects, neurons in the highest network area become tuned to object identity invariant of precise position, comparable to inferotemporal neurons in macaques. Drawing on this, the dynamic properties of invariant object representations reproduce experimentally observed hierarchies of timescales from low to high levels of the ventral processing stream. The predicted faster decorrelation of error-neuron activity compared to representation neurons is of relevance for the experimental search for neural correlates of prediction errors. Lastly, the generative capacity of the network is confirmed by reconstructing specific object images, robust to partial occlusion of the inputs. By learning invariance from temporal continuity within a generative model, the approach generalizes the predictive coding framework to dynamic inputs in a more biologically plausible way than self-supervised networks with non-local error-backpropagation. This was achieved simply by shifting the training paradigm to dynamic inputs, with little change in architecture and learning rule from static input-reconstructing Hebbian predictive coding networks.

## 1. Introduction

How networks of neurons in the brain infer the identity of objects from limited sensory information is one of the preeminent questions of neurobiology. Strengthening theories of generative perception (Gregory, 1980; Mumford, 1992; Rao and Ballard, 1999; Friston, 2010; Pennartz et al., 2019), evidence has accumulated to suggest that the mammalian perceptual system is relying on various forms of prediction to facilitate this process. Across time, repetition suppression that requires explicit expectations (Summerfield et al., 2008; Todorovic et al., 2011), encoding of deviation from temporal expectations in macaque’s inferotemporal and prefrontal cortex (Schwiedrzik and Freiwald, 2017; Bellet et al., 2021) and encoding of expected movement outcomes in mouse V1 (Leinweber et al., 2017) show that the brain constantly tries to predict future inputs. V1 activity evoked by illusory contours (Bartels, 2014; Kok and de Lange, 2014), encoding of information from occluded scene areas in early visual areas of humans (Smith and Muckli, 2010) and modulation of neural responses by expectations based on the surrounding context (Knierim and van Essen, 1992) show that predictions are not only made forward in time, but also across space (in the present). According to predictive coding theory, these predictions are mediated by corticocortical top-down connections (Pennartz et al., 2019) and then corrected based on the received bottom-up input (Rao and Ballard, 1999) in line with hierarchical Bayesian perception (Lee and Mumford, 2003). Predictive coding models have successfully explained properties of the visual system such as end-stopping in V1 neurons and learning of wavelet-like receptive fields (Rao and Ballard, 1999) and V1 activity in illusory contours (Lotter et al., 2020; Pang et al., 2021). However, these studies are focused on low-level effects, while the learned higher-level representations have been investigated much less (although see Dora et al., 2021 for learning of sparse representations).

**Figure 1:**
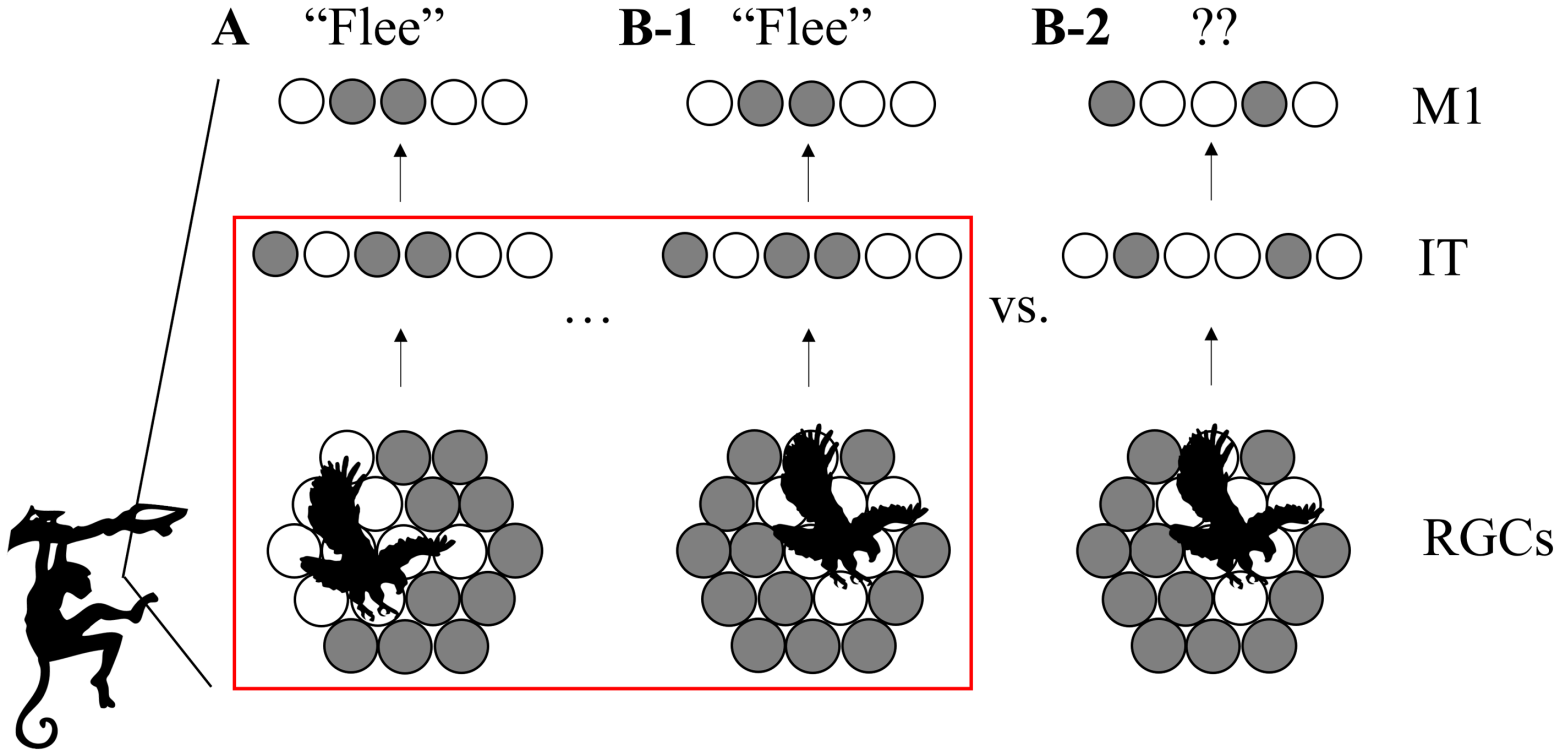
View-invariant representations for efficient cognition. **(A)** Barely escaping an attack, the monkey learns to associate an action (“flee,” encoded by the neural pattern in primary motor area M1) with the activity pattern in its retinal ganglion cells (RGCs, bottom) triggered by the image of an approaching eagle. Active cells are shown in white. **(B-1)** When the monkey later encounters a similar eagle from a different angle, an invariant higher-level representation (center) can still trigger the same action. **(B-2)** Without invariant coding, the action does not generalize to this viewing condition. Red box: scope of this paper: how do multiple low-level activity patterns become linked to one high-level, invariant representation?

Continuously generated by the awake brain, neural representations of the external world form a partial solution to the problem of inference, arguably constituting the basis of conscious experience (Pennartz, 2015), decision-making and adaptive planning (Butz and Kutter, 2016). They can be loosely defined as activity patterns in response to a sensory stimulation elicited by an object. Especially important is the ability to represent multiple views of the same object in similar patterns of activity. These invariant representations have two key advantages: first, information acquired about an object (such as a novel action associated with it) can be linked to only one representation, making learning more efficient. Secondly, as illustrated in Figure 1, the newly acquired invariant information about single objects generalizes automatically across all viewing conditions, facilitating learning from few examples. Evidence for invariant neural representations comes from the ventral temporal lobe (Haxby et al., 2001), the hippocampus in humans (Quiroga et al., 2005), inferotemporal cortex of rhesus (Desimone et al., 1984; Logothetis et al., 1995) and macaque monkeys (Freiwald and Tsao, 2010) as well as rats’ laterolateral extrastriate area (LL) (Tafazoli et al., 2012, 2017). Current theories of how neurons come to acquire such a specialized tuning either fail to account for fundamental aspects of brain circuitry and physiology or rely on artificial learning paradigms. To construct useful representations, biological systems are limited to mostly unsupervised learning (from unlabeled data) and local learning rules, whereas machine vision algorithms based on neural networks typically rely on large amounts of labeled training data and use mechanisms like weight-sharing (LeCun et al., 1989). These mechanisms facilitate generalization across viewing conditions but lack a biological foundation.

A biologically plausible approach to learn view-invariance from transformation sequences is so-called trace learning (Földiák, 1991; Elliffe et al., 2000; Rolls, 2012) which is linked to Slow Feature Analysis (SFA) (Sprekeler et al., 2007). It is based on the idea that temporal proximity between sensory patterns should be reflected in representational similarity, as the assumption can be made about the world that the causes (objects etc.) vary more slowly than the stimulation patterns they evoke on the retina. Indeed there is evidence for the importance of temporal stimulus continuity for learning of transformation-tolerance in early visual areas of rats (Matteucci and Zoccolan, 2020) and area IT of monkeys (Li and DiCarlo, 2008). Based on this principle of representing consecutive inputs similarly Halvagal and Zenke (2022) recently showed that a more intricate learning rule with additional variance maximization leads to disentangled high-level representations. Other self-supervised models avoid representational collapse through contrasting examples (Illing et al., 2021).

However, all of these models process information in a strictly feedforward manner or limit the role of feedback connections to a modulatory function, in contrast to evidence on retinotopic, content-carrying feedback connections in the visual cortex (Zmarz and Keller, 2016; Marques et al., 2018; Pak et al., 2020). Here, we propose a common underlying learning mechanism for both high-level representations and a generative model capable of reconstructing specific sensory inputs: the minimization of local prediction errors through inference and learning.

Like the abovementioned feedforward models of invariance learning, predictive coding offers a mechanism for maintenance of higher-level representations: they are only updated when lower levels send up error signals. It can be implemented in a hierarchical neural network model of the visual processing stream using local, Hebbian learning. Furthermore, it is intimately related to the abovementioned slowness principle, which states that the most meaningful features often change on a slow timescale (Wiskott and Sejnowski, 2002), because extracted causes tend to be good predictors for future input (Creutzig and Sprekeler, 2008). To sum up, predictive coding is a promising candidate to explain learning of invariant object representations within the framework of generative modeling.

To acquire transformation-tolerance from temporal continuity, input sequences are required. Most predictive coding models so far, however, either operate on static inputs (Rao and Ballard, 1999; Spratling, 2017; Dora et al., 2021) or use non-local learning rules (Jiang and Rao, 2022) such as backpropagation (Rumelhart et al., 1985; Singer et al., 2019) and biologically implausible LSTM units (Lotter et al., 2016, 2020). Here, we train multilayered predictive coding networks with only small architectural modifications from Rao and Ballard (1999) and Dora et al. (2021) on transformation sequences with purely Hebbian learning. We confirm learning of a generative model, showing that top-down predictions made by the network approximate the original input. Importantly, these predictions are not forward in time, but across retinotopic space, representing the current input. Presented with partially occluded input sequences, the network pattern-completes the occluded areas through top-down feedback, mimicking functions of human V1 and V2. While reconstructions from lower areas are more faithful, predictive neurons in the network’s higher areas develop view-invariant representations akin to responses of neurons in the inferotemporal area of primate cortex: input stimuli shown in temporal proximity are represented similarly. A decoding analysis confirms that distinct objects are well separable. Lastly, the temporal dynamics of the neural subpopulations are analyzed and compared to recent electrophysiological data from rats. As in the experiment, temporal stability of representation neurons (measured by the decay of autocorrelation) increases as one moves up the hierarchy. In addition, the model makes the prediction that high-level error-coding neurons operate on a faster timescale than their representational counterparts.

## 2. Methods

**Figure 2:**
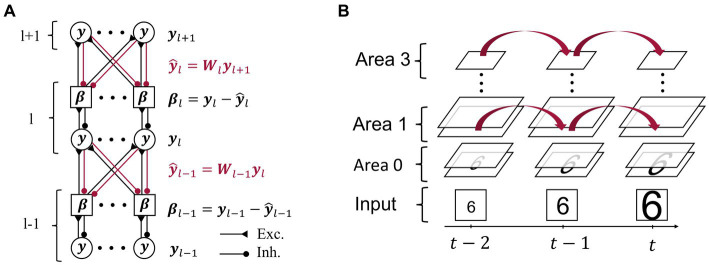
Model architecture and inference on video sequences. **(A)** Representational activity $y_{l}$ in area l (l ∈ {1,2}) is influenced by both top-down predictions highlighted in red via the respective top-down errors $\beta_{l}$ , and by the bottom-up errors $\beta_{l-1}$ . Intra-area connections between representation neurons (circles) and respective error neurons (squares) are one-to-one, while inter-area connections are all-to-all. Synaptic connections between neurons are drawn as filled circles if inhibitory and as triangles if excitatory. **(B)** A sequence of input images is fed into the lowest area of the network across subsequent moments in time (t−2,…,t) . The network maintains representational activity through time and thus uses it as a prior for the inference of subsequent representations.

We developed a neural network consisting of four hierarchically arranged areas. Applying the principles of predictive computation, we restricted ourselves to the minimally necessary components, but other connectivity patterns are conceivable [suggested, e.g., by Heeger (2017)]. As in previous implementations of predictive coding (Rao and Ballard, 1999; Dora et al., 2021), each area contains two subpopulations of neurons that are illustrated in Figure 2:

1. Representation neurons collectively hold the “inferred causes,” in higher areas corresponding to perceptual content. Together with the synaptic connections towards lower areas, they generate top-down predictions to match the current representations in the area below.
2. Error neurons measure the mismatch between representation neuron activity (in the lowest area: the sensory input) and top-down predictions.

Some models such as (Sacramento et al., 2018) suggest computation of errors in dendrites, but based on the evidence for neural encoding of errors (Zmarz and Keller, 2016; Green et al., 2023), we assign dedicated neurons to encode them. Development of such error-tuned neurons has been modeled by Hertäg and Sprekeler (2020) in cortical microcircuits and by Ali et al. (2021) as a result of energy efficiency. While the number of neurons in the input area depends on the dimensions of the dataset and varied between 784 and 1,156, the consecutive areas consisted of [2000, 5,000, 30] neurons (for Area 1, 2, and 3, respectively), except where noted differently. This is supported by an analysis of how altering the number of neurons affects decoding performance in Supplementary material 1.13.

### 2.1. Inference: updating neural activity

At the start of a sequence, all neural activity is set to a uniform, low value (unless stated differently in the Results section). While an image is presented to the network, the lowest area representation neurons linearly reflect the pixel-wise intensity of the input (at the bottom of Figure 2B). Error neurons in area l receive excitatory input from the activity $y_{l}$ of associated representation neurons as shown in the one-to-one connections in Figure 2A, and are inhibited by the summed-up predictions ${\overset{⌢}{y}}_{l}$ from the higher area:


$$\beta_{l}^{\left(t\right)}=y_{l}^{\left(t-1\right)}-{\overset{⌢}{y}}_{l}^{\left(t\right)}=y_{l}^{\left(t-1\right)}-W_{l}^{\left(t-1\right)}y_{l+1}^{\left(t-1\right)}\left(1\right)$$

where bold letters indicate vectors and matrices and $W_{l}^{\left(t-1\right)}$ denotes the symmetric weight matrix between area *l* and area *l + 1* from the previous time step (the weights will change during learning). Strictly symmetric weight matrices as frequently used in predictive coding models (Rao and Ballard, 1999; Dora et al., 2021) lead to a weight transport problem during learning. However, it has been shown that, in combination with weight decay, symmetric weights can be obtained by learning rule comparable to ours without explicitly enforcing symmetry (Alonso and Neftci, 2021), since the locally available pre- and postsynaptic activity that determine the weight change are identical (symmetric) for each pair of feedforward and feedback connections. Each representation neuron receives inhibitory input from one error neuron in the same area and excitatory input from the weighted bottom-up errors and thus changes its activation state at each time step (see “inference” in Alg S1):


$$x_{l}^{\left(t\right)}=x_{l}^{\left(t-1\right)}+\in_{\inf}\left(W_{l-1}^{{\left(t-1\right)}^{T}}\beta_{l-1}^{\left(t\right)}-\beta_{l}^{\left(t\right)}\right)\;\;\;\;\;\;\;\;\;\;\;\;\;\;\;\;(2)$$

This adjustment of neural activation state $x_{l}$ (akin to membrane potential) of representation neurons can be interpreted as matching top-down predictions better than before (and thus reducing activity of the associated error neuron) and sending down predictions that better match representation neuron activity in the area below (thus reducing errors there). The rate at which neuronal activation is changed is governed by the parameter $\epsilon_{\inf}=0.05$ referred to in the following as the *inference rate*. The activation state $x_{l}$ is now translated into an output firing rate $y_{l}$ :


$$y_{l}^{\left(t\right)}=\phi \left(x_{l}^{\left(t\right)}+\Delta_{x}\right)\left(3\right)$$

where ϕ denotes the sigmoid activation function, and $\Delta_{x}$ a constant lateral offset of the firing threshold. The saturation of the sigmoid for large inputs corresponds to a maximal firing rate of the representation neurons, in contrast to the more artificial (rectified) linear activation functions used in Rao and Ballard (1999) and Dora et al. (2021) that do not have an upper bound.

### 2.2. Learning without labels: updating synaptic strengths

Before training, weights are initialized to random values from a Gaussian distribution centered at zero and with standard deviation of 0.5, clipped at zero to prevent negative weights and divided by the number of neurons in the next (higher) area. After 10 inference steps, long-term adaptation of synaptic weights is conducted in a Hebbian manner, strengthening synapses between active error neurons in area *l* and simultaneously active representation neurons in the area above (*l + 1*):


$$W_{l-1}^{\left(t\right)}=W_{l-1}^{\left(t-1\right)}+\in_{learn}\cdot \beta_{l-1}^{\left(t\right)}y_{l}^{\left(t\right)}{}^{T}\;\;\;\;\;\;\;\;\;\;\;\;\left(4\right)$$

with learning rate $\epsilon_{learn}$ . Apart from not using weight decay, normalization or a gating mechanism, we thus use the same learning rule as Rao and Ballard (1999) and Dora et al. (2021). Based on the slower change of synaptic efficacy in comparison to membrane potential dynamics, weights are assumed to be constant between these updates. In Equation 4 , the sign of the prediction error controls the direction of the weight change. If the prediction is too large relative to the activity of the representation neurons in this area, the error is negative, and the weight mediating the prediction will be reduced. As a result, given the same prediction, the error in the consecutive time step will be smaller. This stabilizing effect on the response of error neurons is familiar from the work of Vogels et al. (2011) that showed how Hebbian plasticity regulates inhibitory input to reduce firing and achieve a balanced global state.

To summarize, both the balanced, excitatory-inhibitory wiring of the network and the unsupervised adaptation of weights based on remaining prediction errors lead to an alignment of representations and predictions, and thus a reduction in error neuron activity. The sum of squared prediction errors can then be seen as an implicit objective function, upon which the inference steps conduct an approximate gradient descent taking into account only the sign and not value of the derivative of the activation function, unlike (Whittington and Bogacz, 2017), and upon which learning conducts a precise gradient descent.

### 2.3. Training procedure

**Figure 3:**
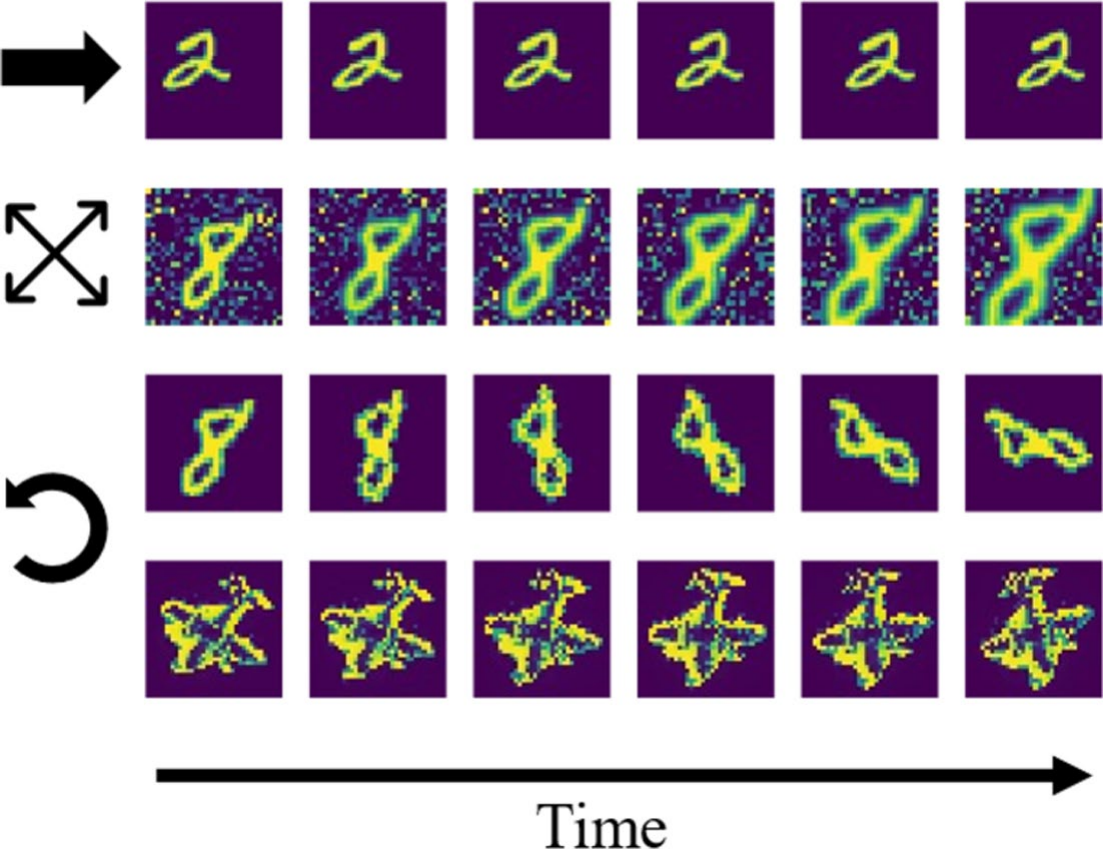
Example sequences from the stimulus datasets. First row: digit translation (indicated by the horizontal arrow) without noise. Second row: digit scaling (expanding arrows) with noise. Third and fourth row: rotation (indicated by the counterclockwise arrow) of digit/toy plane without noise.

We trained the network on temporally dynamic inputs, using short video sequences. After validating network performance on moving horizontal and vertical bars, we switched to using 10 digits of the MNIST handwritten digits dataset (one per digit from 0 to 9). Each sequence contained six gradually transformed images, and separate datasets were created for translational motion, rotation, and scaling (Figure 3). For translational and rotational motion, two transformation speeds were used, differing in overlap between consecutive images. The examples shown in Figure 3 are from the dataset with larger step size (“fast” condition). To further examine robustness of the training paradigm under more realistic and less sparse inputs, random noise patterns were added to the image background during training. A last dataset consisted of five high-pass filtered images of toy objects (an airplane shown in the last row of Figure 3, a sports car, a truck, a lion and a tin man) from the smallNORB dataset (LeCun et al., 2004), undergoing a rotation.

The network was trained on the 10 (for the toy objects: five) sequences, each presenting a different digit, for multiple epochs. Each epoch consisted of 10 iterations of the same sequence (e.g., of a moving digit ‘6’) before switching to the next (of digit ‘7’). All hyperparameters are summarized in Supplementary material 1.1. This repetition of individual sequences drastically improved network performance and could be achieved by the brain through a replay or reactivation mechanism (observed in visual cortex by Ji and Wilson, 2007 and Xu et al., 2012, see also Wilson and McNaughton, 1994; Lansink et al., 2009). For laterally moving stimuli, repeated presentation can also be achieved by object-tracking saccades that lead to repeated motion across the same photoreceptors on the retina. As the most information-neutral state, the activity was reset to uniform, low values at the beginning of each sequence. This assumption is justified for objects that are seen independently of each other; for instance, not every ‘6’ is followed by a ‘7’ (but see Supplementary material 1.15 for how this assumption can be relaxed). For each image, multiple inference-learning cycles (Equation 1–4) were conducted before switching to the next image in the sequence. A training epoch consisted of an iteration through all sequences from the dataset. Supplementary material 1.2 contains the pseudocode for the nested training loops.

### 2.4. Analysis of neural representations

To quantify to what extent the network learned representations that are invariant to transformation, while at the same time retaining meaningful information about sample identity, we combined representational similarity analysis (Kriegeskorte et al., 2008) with linear decoding. The distance *d* between two representations $r_{\;1}$ and $r_{2}$ , vectors of neural activity in a model area, was measured via cosine dissimilarity:


$$d\left(r_{1},r_{2}\right)=1-\frac{r_{1}\cdot r_{2}}{∥r_{1}∥\;\;∥r_{2}∥}\left(5\right)$$

Linear decoding was conducted by mapping the inferred representations through a fully connected layer to a layer with one neuron per class label. We implemented this via the linear model class and fitting function of the sklearn library in Python.^1^ Decodability was then measured by the classification accuracy on representations that the decoder had not been presented with before. How well the decoder generalized from representations of a subset of samples from each sequence to the other views of the object is a direct measure of downstream usefulness in the scenario outlined in Figure 1.

## 3. Results

We trained the network on sequences of moving objects as specified in the Methods section, and focused on the evolving high-level representations, resulting neural dynamics, and generative input-reconstructing capacities of the network, all in comparison to neurobiology.

### 3.1. Transformation-invariant stimulus representations

**Figure 4:**
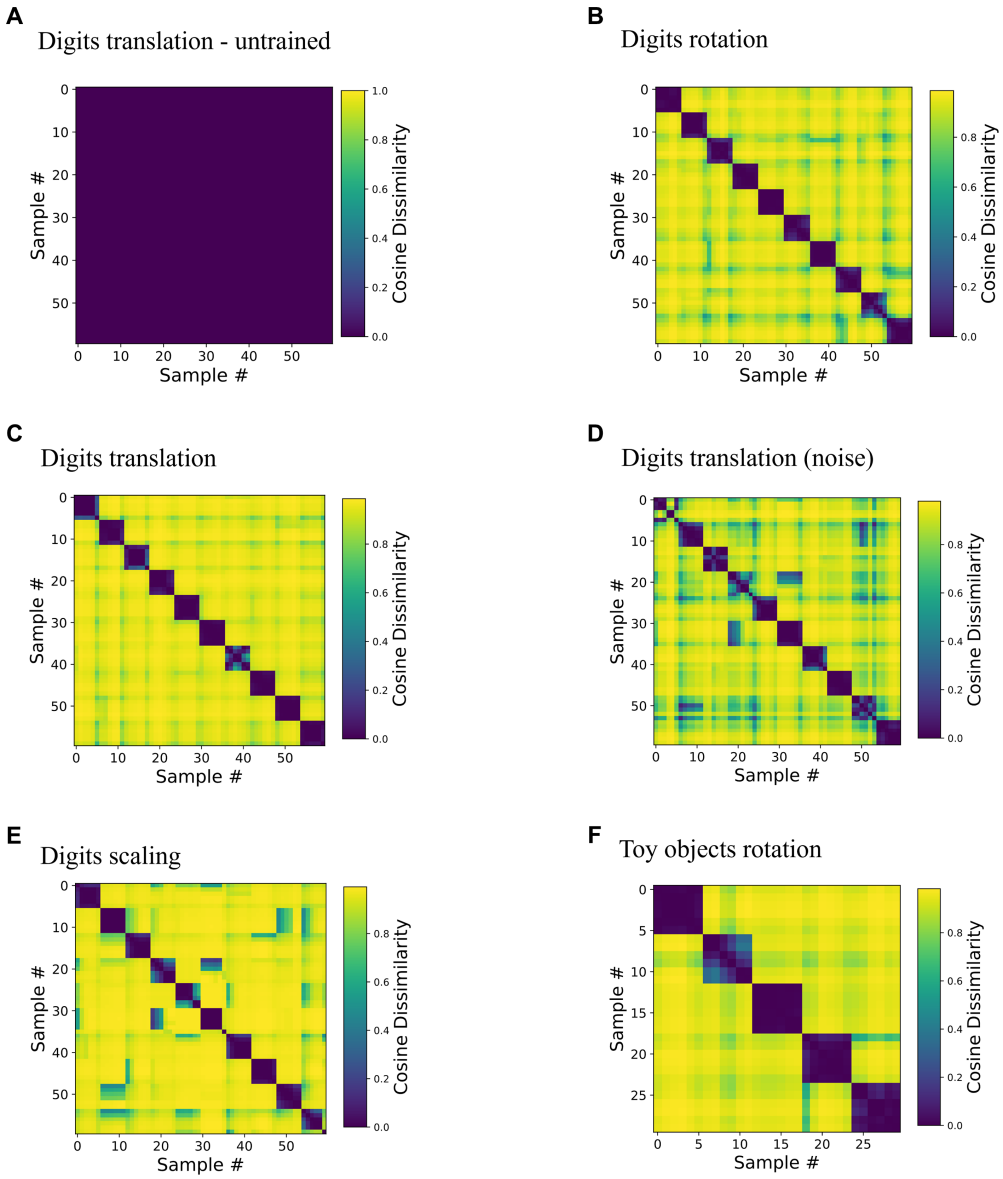
Representations invariant to viewing conditions are learned without data labels. The matrices depict cosine dissimilarity between representations in area 3. Each of the rows and columns in these plots corresponds to one input image (i.e., a digit sample in a specific spatial configuration), thus each matrix is symmetrical. Along each dimension, samples are ordered sequence-wise, i.e., rows and columns 0–5, 6–11 etc. are the same object in six different transformation states. Low values shown in purple correspond to similar activity patterns, i.e., a similar set of neurons represents the stimuli given by the combination of row and column, high values shown in yellow correspond to orthogonal activity vectors. **(A)** Baseline, an untrained network tested on the translationally moving digits dataset, for untrained versions of the other RDMs see Supplementary Figure S8. **(B–E)** Networks trained and tested on one of the three datasets of ten rotating, translating (with and without noise) and scaling digits show a clear block-diagonal structure with low values for comparisons within sequences. **(F)** Network trained and tested on five rotation sequences of toy objects.

We found that neurons in network area 3 became tuned to samples in a position-invariant manner. To quantify invariance, we analyzed the neural representations in the highest area of trained networks (Figure 2) under changes of inputs. More specifically, inference was run on still images from the training datasets until convergence was reached (see Supplementary material 1.3 for a description of convergence). Then, pairwise comparison of inferred area 3 representations measured in cosine distance quantified representational dissimilarity between representations of the same sample, e.g., a digit (within-sequence) or different samples (across-sequence). All pairwise values were plotted in Representational Dissimilarity Matrices (RDMs, Kriegeskorte et al., 2008) in Figure 4.

Indicating invariance, RDMs of trained networks showed high similarity within sequences; for instance, Digit “1”, “2”, etc. was represented by highly similar activity patterns in area 3, irrespective of position. Representations of samples from different sequences, such as digit “1” and digit “2” at the same position were distinct, as indicated by a high dissimilarity in matrix elements off the block diagonal. The same held true for the rotating and scaling digits (Figures 4B,E) as well as for the five rotating toy objects (Figure 4F). Supplementary material 1.9 contains a proof of principle demonstration of learning multiple transformations in the same network. Noise (shown for the translational motion in Figure 4D versus the noiseless motion in Figure 4C) slightly degraded clarity of the RDM but preserved the overall structure well. Additionally, the structure of the RDM proved to be quite tolerant to smaller weight initialization (Supplementary material 1.7).

Invariance of representations was a consequence of learning from inputs that are transformed continuously in the temporal domain as evidenced by the RDMs of the untrained network that showed very little structure (Figure 4A, note the different color scale, cosine distance below 0.001). Networks trained on the static frames of the sequences, in which activity was reset after each frame also lacked a block-diagonal structure (Supplementary Figure S2), illustrating the role of continuous motion in the training paradigm, which is to provide the necessary temporal structure in which subsequent inputs can be assumed to be caused by the same objects. Interestingly, we did not find an influence of sequence order on decoding accuracy (Supplementary Figure S11), suggesting that only temporal (as shown by the comparison to the static training paradigm), but not spatial continuity of the input transformations was necessary for successful representation learning. The Hebbian learning rule thus groups together consecutive inputs in a manner reminiscent of contrastive, self-supervised methods (Van Den Oord et al., 2019; Illing et al., 2021; Halvagal and Zenke, 2022) that explicitly penalize dissimilarity in the loss function. Here, the higher-level representation from the previous timestep provides a target for the consecutive inputs reminiscent of implementations of supervised learning with local learning rules (Lee et al., 2015; Whittington and Bogacz, 2017; Haider et al., 2021).

**Figure 5:**
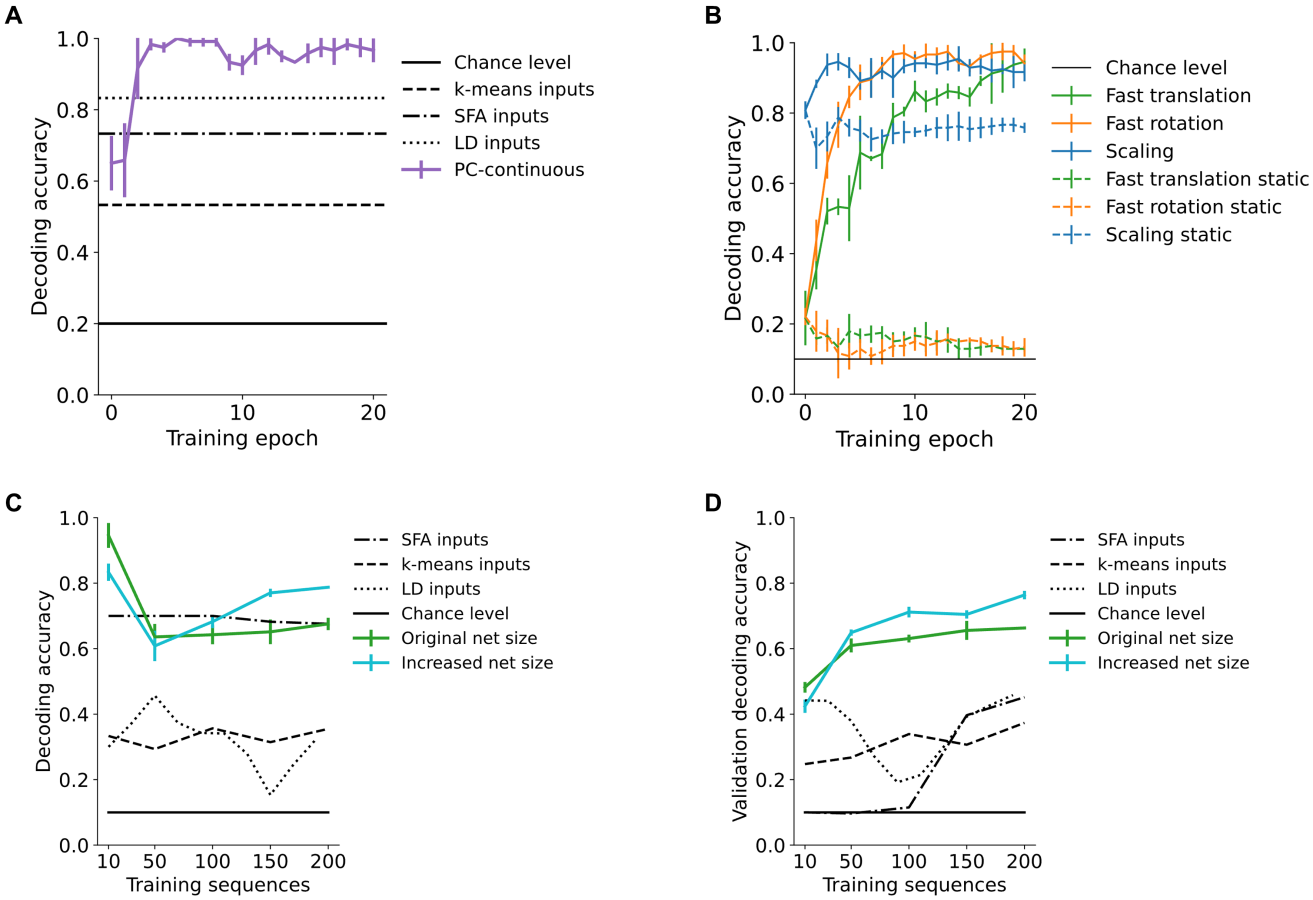
High-area representations encode object identity. Decoding accuracy of a linear decoder operating on area 3-representations of our predictive coding network trained in the continuous paradigm (PC-continuous) plotted across training epochs (iterations through the whole dataset). **(A)** Accuracy quickly rises above performance of k-means clustering, SFA and linear decoding directly on the input data (LD inputs) for the rotating toy objects dataset. The error bars for all figures are computed across four random seeds for the weight initializations. **(B)** Influence of continuous training: decoding accuracy in networks trained on continuous sequences (continuous lines) is increased compared to networks trained on isolated (static) frames of the sequences. **(C)** When increasing the size of the dataset from 10 to 200 sequences, the network of original size maintains a decoding accuracy far above chance level. Here, accuracy is significantly improved when the number of neurons in [area 1, area 2, area 3] is increased from [2000, 500, 30] (green curve) to [4,000, 2000, 90] neurons (blue curve). **(D)** Decoder accuracy on a previously unseen validation set of 200 randomly selected and transformed digits.

Area 3-representations were informative about the identity of the sample moving in sequence as decodability improved with training (Figures 5A,B). In addition to its behavioral relevance, decodability of representations quantifies the learned within-sequence invariance. A biologically plausible way to make high-level object representations available to downstream processes (such as action selection, Figure 1) is a layer of weighted synaptic connections, i.e., a linear decoder, to infer object identity. We simulated this through a linear mapping of the converged area 3-activity vectors that were obtained as above to 10 object identity-encoding neurons (digits “0”, “1”, …, “9”). After fitting the decoding model to 2/3 of the representations, evaluation was conducted on the remaining 1/3 in a stratified k-fold manner (with *k* = 3). Compared to the information content in the input signal, as measured by the accuracy of a linear decoder, as well as k-means clustering, area 3 representations achieved better decoding performance after around five training epochs (Figure 5A). The model also outperformed linear Slow Feature Analysis (SFA) (Wiskott and Sejnowski, 2002) of the raw inputs (for details see Supplementary material 1.12). This was confirmed across almost all used datasets (Table 1) and even increased as the transformation step size was increased, resulting in smaller overlap between consecutive images (“fast” conditions in Table 1, shown in the first and third row of Figure 3). Across the hierarchy, higher network areas developed more invariant representations than lower areas (Supplementary Figure S9).

**Table 1:** Decoding accuracy (in percent) across datasets and models.

Dataset	PC-continuous, area 3	PC-static, area 3	*k*-means input	Linear decoding input	SFA input
Toy objects rotating	**95.83 ± 5.46**	18.33 **±** 1.67	53.33	83.33	73.33
Digits rotating	**94.17 ± 2.50**	13.33 **±** 2.64	48.33	85.00	70.00
Digits rotating, noise	**90.00 ± 5.14**	15.83 **±** 1.86	N/A	N/A	N/A
Digits scaling	91.67 ± 2.64	75.83 **±** 1.44	68.33	**100.00**	70.00
Digits scaling, noise	**86.67 ± 4.25**	71.25 **±** 12.33	N/A	N/A	N/A
Digits translation	**83.75 ± 6.50**	40.42 **±** 4.31	60.00	75,00	70.00
Digits translation, noise	**93.33 ± 4.08**	47.92 **±** 6.60	N/A	N/A	N/A
Digits fast rotation	**94.17 ± 2.50**	13.33 **±** 2.64	31.67	40.00	70.00
Digits fast translation	**94.58 ± 3.80**	12.92 **±** 0.72	38.33	25.00	70.00
Digits fast translation, noise	**87.50 ± 4.49**	14.17 **±** 3.00	N/A	N/A	N/A

The best performing decoder per dataset is marked in bold. The left column is the predictive coding network trained in the continuous manner put forward in this paper.

Decodability of network representations was maintained when the dataset size was significantly increased. We tested this by training networks on up to 20 random digits per digit class (totaling 200 sequences of the fast translations). As shown in Figure 5C, the network maintained above 60% linear decoding accuracy of digit class while an enlarged version of the network shown in cyan further improved this. On the other hand, increasing dataset size negatively affected the invariance structure of the RDMs (Supplementary material 1.8). putatively due to the limitations discussed in section 4.4.

Lastly, generalization performance for the remaining MNIST dataset was measured by decoding accuracy on previously unseen digits. Here, accuracy was above 60% when more than 100 training sequences were used (Figure 5D). In the enlarged network, decoding accuracy rose above 75% (the blue line in Figure 5D), confirming the network’s capacity to generalize. The small standard deviation between randomly initialized runs indicates the representativeness of the chosen validation subset.

The continuous training paradigm improved decoding performance in comparison to networks trained on static inputs. There, decoding performance dropped from the initial value and was consistently more than 20 percentage points worse than in the continuously trained network (Figure 5B and Table 1). This can partially be explained by the learning of more sample-specific and thus less invariant representations in the static training paradigm, where activity was not carried over from one image to the next (Supplementary Figure S2).

### 3.2. Temporal stability of representations

**Figure 6:**
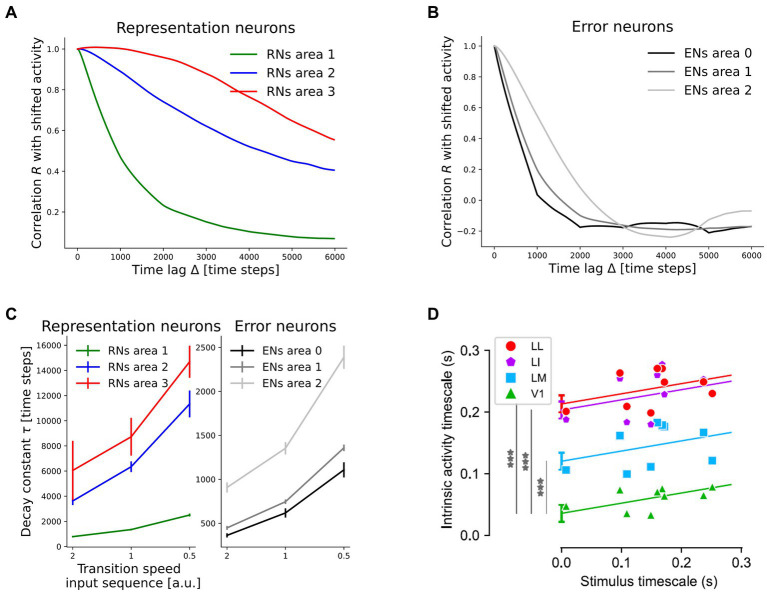
The network develops a hierarchy of timescales comparable to experimental data from rodent visual cortex. **(A)** Temporal decay of activity autocorrelation for the representation neuron subpopulations (RNs). **(B)** Decay of autocorrelation for the error-coding subpopulations (ENs). **(C)** Inferred decay constants per subpopulation across stimulus timescales. Error bars denote one standard deviation across four randomly initialized networks (*p*-values are given in the main text). Increasing the speed by a factor of two corresponds to 50% less inference steps per frame. **(D)** Comparison to experimental evidence from rat visual cortex. Hierarchical ordering of intrinsic timescales rendered as the decay constants of activity autocorrelation, from V1 across lateromedial (LM), laterointermediate (LI) to laterolateral (LL) visual areas, adapted from Piasini et al. (2021) under the license https://creativecommons.org/licenses/by/4.0. ***p = 5e-7, 1e-13, 2e-14, respectively for LM, LI, and LL.

Without explicitly integrated constraints, the network developed a hierarchy of timescales in which representations in higher network areas decorrelated more slowly over inference time than in lower areas. We quantified this by measuring the autocorrelation *R* during presentation of rotating digits. It is defined as


$$R\left(z,\Delta \right)=\frac{1}{N\left(T-\Delta \right)}\overset{T-\Delta }{\underset{t=1}{\sum }}z\left(t\right)\cdot z\left(t+\Delta \right)\;\left(6\right)$$

where ∆ is the time lag measured in inference steps between the points to be compared, *T* is the duration of each sequence (consisting of 6,000 inference steps), *N* the number of neurons in the subpopulation and **z** (t) the activity vector in the subpopulation (averaged across 10 inference steps). High values indicate similar, non-zero activities and thus high temporal stability. The resulting autocorrelation curves for time lags between 0 and the length of an individual sequence are shown in Figures 6A,B, averaged across the 10 rotation sequences. From these curves, decay constants were inferred by measuring the time until decay to 1/e. If that value was not reached until the sequence end, we extrapolated by using a linear continuation through the values at Δ = 0 and Δ = 6,000 time steps. Additionally, we varied the stimulus timescale by dividing the number of inference steps on each frame by the rotation speed. The resulting decay constants showed a clear and robust hierarchy across network areas, as well as a positive correlation with the stimulus timescale (Figure 6C). A significant difference was found between representation neurons in area 3 and area 1 (mean difference at speed one: 7377 time steps, *p* = 1.61e-2). *p*-values were determined by a Games-Howell post-hoc test (an extension of the familiar Tukey post-hoc test that does not assume equal variances) succeeding rejection of the null hypothesis across the six populations in a Welch’s ANOVA, as described in more detail in Supplementary material 1.16. A smaller, but significant difference was observed between representation neurons in area 2 and area 1 (4,996 time steps, *p* = 9.68e-4). In error-coding neurons, the hierarchy was less pronounced, but area 0 and area 2 nonetheless showed a significant difference (*p* = 9.50e-5). Comparison to a statically trained network with the same architecture which failed to develop a temporal hierarchy in representations (Supplementary material 1.4) showed that the temporal hierarchy was not built into the model architecture, but instead is an emergent property of the model under the continuous training paradigm. This is underlined by the fact that the same inference rate was used in all network areas. The hierarchy in representational dynamics as well as the positive correlation with stimulus dynamics is in agreement with experimental findings in rat visual cortex (Piasini et al., 2021). There, the authors computed neuronal timescales for the decay of autocorrelation in a similar manner and found more stable activity patterns in higher areas of rat visual cortex (Figure 6D).

The decay speed of autocorrelation also allowed us to differentiate between quickly decorrelating error neurons and more persistent representation neurons in higher network areas. Error-coding neurons in area 2 showed a shorter activity timescale than representation neurons within the same area. The difference equaled 4,991 time steps (*p* = 9.88e-4), compared to only 211 time steps in the statically trained network (Supplementary Figure S3). In this context Piasini et al. (2021) discussed the following scenario: when perceiving a continuously moving object, its identity is predictable over time. Thus, one could expect a diminishing firing rate in neurons representing this object, in contrast to their evidence on larger timescales in higher visual areas. Our results reconcile the framework of predictive coding with these empirical observations by differentiating between quickly decorrelating error-signals and persistent representations. Remarkably, this prediction about the consequences of predictive coding circuitry for the activity autocorrelation timescales of error- and representation neurons has, to our knowledge, not been proposed before. Here it is important to mention the extensive literature on the analysis of different frequency bands in cortical feedforward and feedback signal propagation [summarized in Bastos et al. (2012) from a predictive coding perspective]. These sources did, however, not speak about temporal stability and the two concepts are not easily connected. It is, for instance, conceivable to have low-frequency signals that quickly decorrelate or high-frequency signals that are maintained over time.

### 3.3. Generative capacity

**Figure 7:**
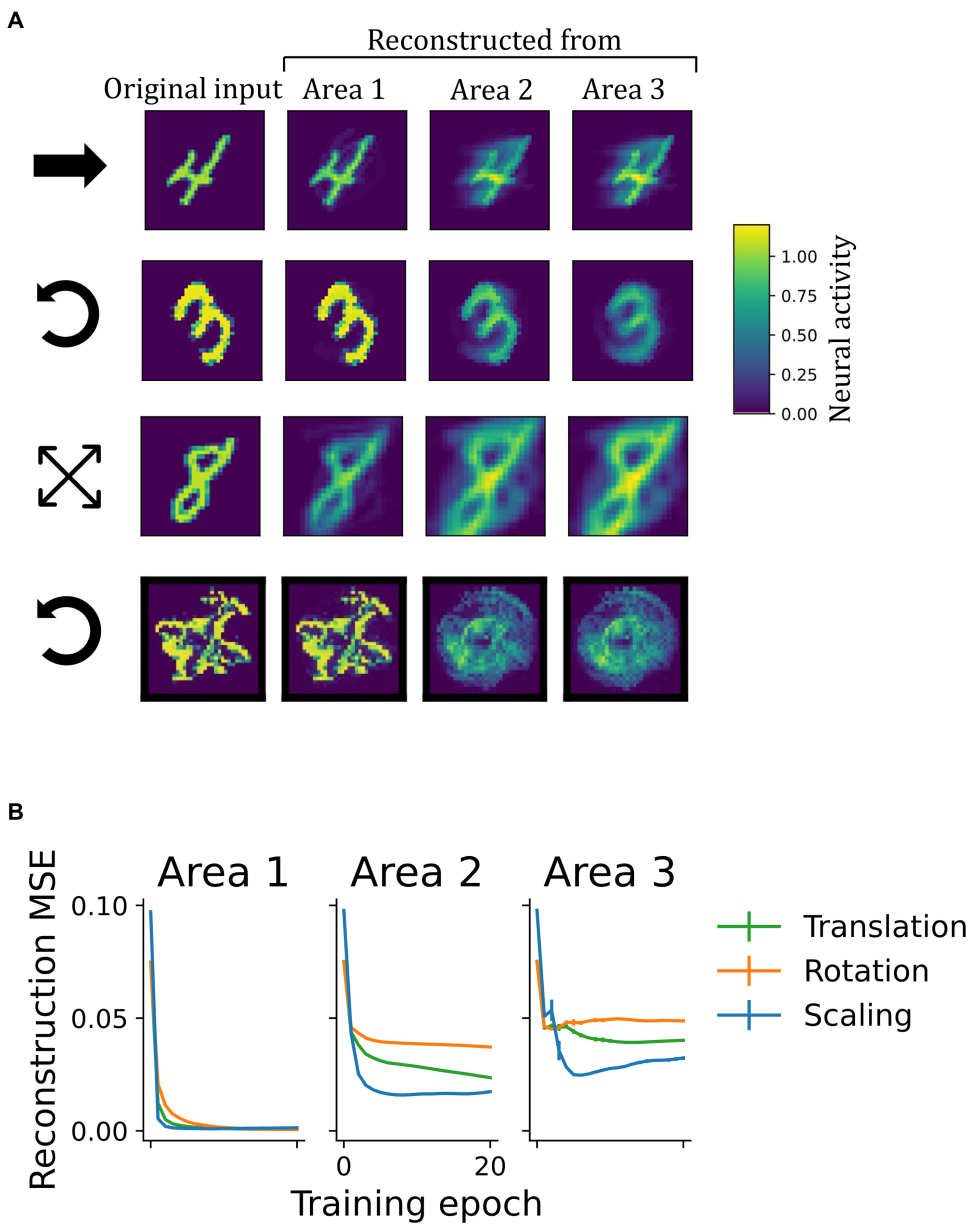
Learning of a generative model. **(A)** Illustration of top-down reconstructions in the model with invariant representations. The first column depicts original input images from different datasets. Columns two to four show the activity pattern in the input area generated by propagating latent representations from different network areas to the input layer in a top-down manner. The symbols at the beginning of each row indicate the underlying transformation: translation, rotation, scaling and rotation, respecitvely (as in Figure 3). In early network areas, representations inferred from sensory inputs carried enough information to reconstruct the input image once it was removed. Reconstructions from higher areas were less accurate. **(B)** Mean squared reconstruction errors (MSE), comparing the original input to the reconstructions on a pixel-level. The vanishingly small vertical bars indicate the standard deviation across four random seeds.

The network learned a generative model of the visual inputs as shown by successful input-reconstruction through the network’s top-down pathway (Figure 7). Since areas further up in the ventral processing stream of the cerebral cortex are thought to encode object identity, it is interesting to ask, in how far they are to be able to encode fully detailed scene information, or whether they contain only reduced information (such as object identity). To examine the functioning of this reverse pathway under the continuous transformation training paradigm, we investigated the representational content in each area by reconstructing sensory inputs in a top-down manner. After training, a static input image was presented until network activity converged (Supplementary material 1.3). Then, the input was blanked out and the inferred activity pattern (representation) from a selected area was propagated back down to the input neurons via the top-down weights. Area by area, activity $y_{l}$ of representation neurons was installed by the descending predictions ${\overset{⌢}{y}}_{l}$ (see Supplementary material 1.5 for details).

As shown in Figure 7A, the accuracy of reconstruction strongly depended on the area it was initiated from. While predictions from latent representations in area *1* gave rise to reconstructions that resembled the original inputs and achieved low reconstruction errors (Figure 7B), higher areas were less accurate. From there, reconstructions were either blurry or showed the stimulus in a different position, rotational angle, or scale than presented prior to construction (e.g., the “0” from area 3 in the second row of Figure 7). This logically follows from the invariance achieved in these higher areas, from where a single generalized representation cannot suffice to regenerate many specific images. Despite this limitation in obtaining precise reconstructions, which resulted from training on extended sequences instead of individual frames, area 1-representation neurons in all networks contained enough information to regenerate the inputs, thus confirming that the model had learned a generative model of the dataset.

### 3.4. Reconstructing objects from occluded scenes

**Figure 8:**
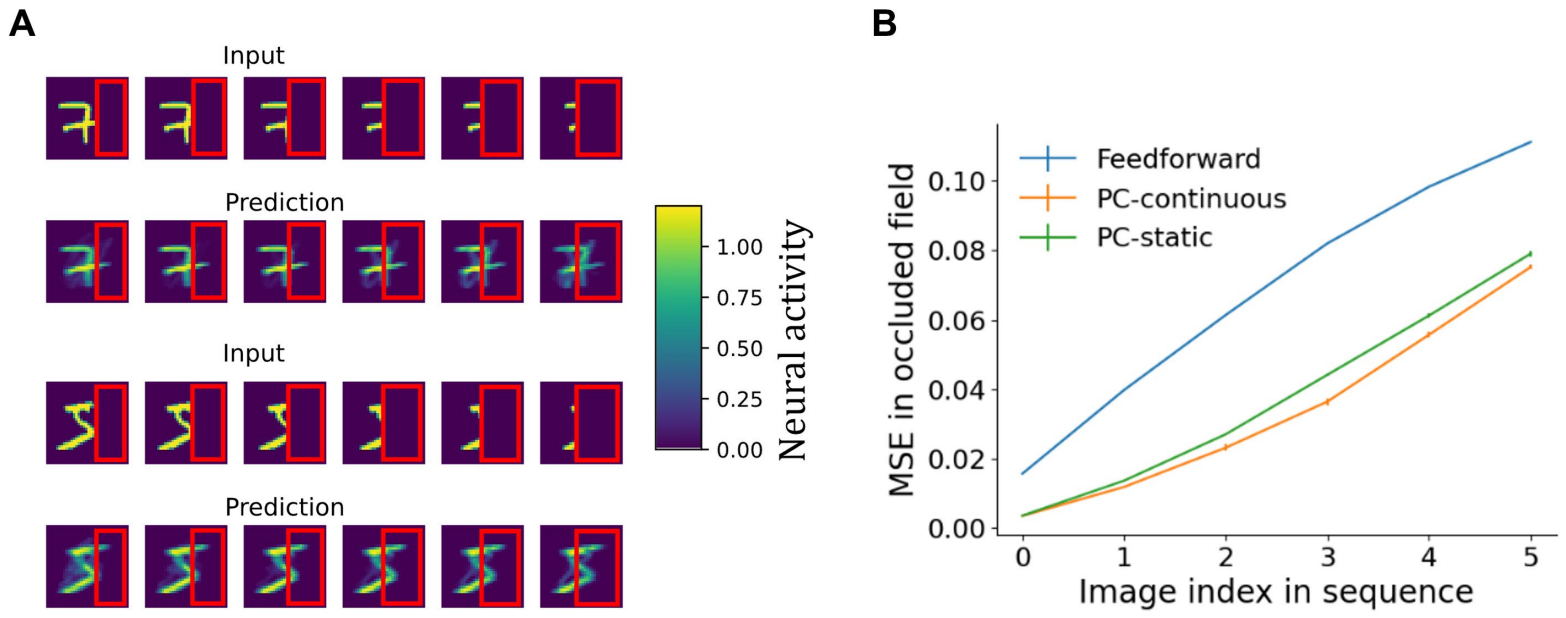
Reconstruction of partially occluded sequences. **(A)** First and third row: the input sequences shown to the PC-continuous network with the occluder outlined in red. Rows two and four: images arising from top-down predictions sent to the area 0 carried information about occluded areas of the input. **(B)** Comparison of a continuously trained predictive coding network to a purely feedforward network (no reconstruction) and a predictive coding network trained on static images. Shown is the mean squared reconstruction error in the occluded part, averaged across all ten sequences, rising as the occluded field becomes larger (plotted over the first to last image of the occlusion sequence). The vanishingly small error bars indicate the standard deviation across four network initializations.

The generative capacity of the network’s top-down pathway was further confirmed by its ability to reconstruct whole objects from partially occluded sequences as shown in Figure 8. A behaviorally relevant use of a generative pathway is the ability to fill in for missing information, such as when guessing what the whole scene may look like and planning an action toward occluded parts of an object. To investigate filling-in in the model, we presented occluded test sequences to the network trained on laterally moving digits (the same as before). After inference on each frame of the test dataset, the predictions sent down to the lowest network area were normalized and plotted retinotopically in Figure 8. Details on the reconstruction process can be found in Supplementary material 1.6. Indeed, predictions sent toward the lowest area carried information about the occluded parts (Figure 8A).

As the input deteriorated, predictions also visibly degraded, resulting in a rising MSE (Figure 8B). The continuously trained network consistently achieved slightly, but significantly better reconstructions than its counterpart trained on static images (for a more detailed analysis see Supplementary material 1.6). An independent t-test resulted in *p* < 6e-4 for all sequence frames except for the first, unoccluded frame where the difference was non-significant. That the difference was small can be explained by two opposing mechanisms: on the one hand memorization of specific frames putatively aids reconstruction in the static network (Supplementary Figure S2). On the other hand, availability of invariant object identity from the temporal context, which can be expected to improve reconstruction in the continuously trained network. Overall, the availability of top-down information in occluded fields of network area 0 is comparable to the presence of concealed scene information observed in early visual areas of humans (Smith and Muckli, 2010) that cannot be explained by purely feedforward models of perception. Unlike auto-associative models of sequential pattern-completion (Herz et al., 1989), our network forms hierarchical representations comparable to Illing et al. (2021).

## 4. Discussion

### 4.1. Summary of results

We have shown how networks that minimize local prediction errors learn object representations invariant to the precise viewing conditions in higher network areas (Figure 4), while acquiring a generative model in which especially lower areas are able to reconstruct specific inputs (Figures 7, 8). The learned high-level representations distinguish between different objects, as linear decoding accuracy of object identity was high (Figure 5). Comparison to considerably worse decoding performance in networks trained on static images underlined the importance of temporally continuous transformation for the learning process (Figure 5A), noting that spatially ordered sequences (as in, e.g., visual object motion) are not strictly necessary (Supplementary Figure S11). Focusing on the implications for neural dynamics, learning from temporally continuous transformations such as continuous motion led to a hierarchy of timescales in representation neurons that showed more slowly changing activity in higher areas, where they notably differed from the more quickly varying error neurons (Figure 6).

### 4.2. A generative model to learn invariant representations

Without the need for explicit data labels, the model developed meaningful, decodable representations purely by Hebbian learning. Linking slowly varying predictions in higher areas to more quickly changing inputs in lower areas lead to emergence of temporally stable representations without the need for an explicit constraint for slowness as used for example in Wiskott and Sejnowski (2002). At the same time, the model acquired generative capacity that enables reconstruction of partially occluded stimuli, in line with retinotopic and content-carrying feedback connections to V1 (Smith and Muckli, 2010; Marques et al., 2018), see also (Pennartz et al., 2019) for a review of predictive feedback mechanisms. Other neuron-level models of invariance-learning (LeCun et al., 1989; Földiák, 1991; Rolls, 2012; Halvagal and Zenke, 2022) neither account for such feedback nor experimentally observed explicit encoding of mismatch between prediction and observation (Zmarz and Keller, 2016; Leinweber et al., 2017) and used considerably more complex learning rules requiring a larger set of assumptions (Halvagal and Zenke, 2022). Conversely, auto-associative Hopfield-type models that learn dynamic pattern completion from local learning rules (Herz et al., 1989; Brea et al., 2013) do not learn hierarchical invariant representations like the proposed model does. By solving the task of invariance learning in agreement with the generativity of sensory cortical systems, the claim for predictive coding circuits as fundamental building blocks of the brain’s perceptual pathways is strengthened.

### 4.3. Related work

We argue that the model generalizes predictive coding to moving stimuli in a biologically more plausible way than other approaches (Lotter et al., 2016, 2020; Ali et al., 2021) that rely on error backpropagation, which is non-local (Rumelhart et al., 1985) or the equivalently non-local backpropagation through time (BPTT, Ali et al., 2021). BPTT achieves global gradient descent and thus generally offers performance benefits over Hebbian learning rules. However, it is not straightforward to combine BPTT with invariance learning from temporal structure and direct comparison is thus difficult. As our network is based on the principles developed by Rao and Ballard (1999), its basic neural circuitry is shared with other implementations of predictive coding with local learning rules derived from it Whittington and Bogacz (2017) and Dora et al. (2021). In terms of scope of the current model, focusing on representational invariance and investigating the consequences of training on dynamic inputs clearly distinguishes the present approach from Dora et al. (2021). Mechanistic differences are biologically motivated, such as omissionof a gating term used by Dora et al. (2021) that depended on the partial derivative with respect to presynaptic neuronal activity. This minimizes the set of necessary assumptions compared to other implementations that require such a term in inference (Whittington and Bogacz, 2017; Dora et al., 2021) and/or learning (Dora et al., 2021). Unlike (Dora et al., 2021), the present implementation also does not require weight regularization that depends on information not readily available at the synapses.

### 4.4. Limitations in performance

Although sufficient for learning of invariant representations on the datasets considered here, the fully connected architecture we used can be expected to limit the degree of representation invariance (as visible, e.g., in the structure of the RDMs) for more complex datasets. However, it has been shown that the lack of inductive bias in fully connected models can be compensated for by training on larger amounts of data (Bachmann et al., 2023). Here, the self-supervised nature of our model is an advantage, as it does not require labeled data. Another interesting extension of the model will be to investigate other common types of transformation such as rotation of three-dimensional objects into the plane. Based on the model’s ability to deal with the scaling transformation and 3D toy objects, we do not expect any fundamental obstacle: the temporal structure of the transformation is important, not the way that it affects the image.

Fully connected areas may also restrict performance on out-of-sample testing. Here, combination of receptive field-like local filters with a pooling mechanism (Riesenhuber and Poggio, 1999) may be helpful to become tolerant to the varying configurations of individual features comprising the objects from the same class. Using a weakly supervised paradigm could improve decoding accuracy even further. It has been shown that under constraints which would be out of the scope of this paper to discuss, inversely connected predictive coding networks can do exact backpropagation when clamping the highest layer activities in a supervised manner (Whittington and Bogacz, 2017; Salvatori et al., 2021).

Input reconstructions from higher network areas degraded as representations became more invariant. This is a direct consequence of Equation 7: each element from the set of area 3-representations casts a unique prediction to the area below. Consequently, multiple different (not invariant) area 3 patterns would be necessary to fully reconstruct a sequence of inputs. Thus, either the invariance in area 3 or the faithfulness of the reconstruction suffers. Nevertheless, the network as a whole appeared to strike a good balance in the trade-off of memorizing information to reconstruct individual samples in lower areas (hence the better reconstruction accuracy from area 1 in Figure 7) and abstracting over the sequence, where area 3 represents object identity invariantly (Figure 5), fitting theoretical descriptions of multilevel perception (ch. 9 in Pennartz, 2015). The more detailed and sample-specific information may provide useful input to the action-oriented dorsal processing stream (Goodale and Milner, 1992), whereas the hierarchy of the ventral visual cortex extracts object identity and relevant concepts (Mishkin et al., 1983).

### 4.5. Hypotheses on the neural circuitry of predictive coding

The model captures neural response properties in early and high-level areas of the visual cortical hierarchy. Retinotopic (Marques et al., 2018) and information carrying (Smith and Muckli, 2010) feedback to early visual areas (cf. Figures 7, 8) as well as invariant (Logothetis et al., 1995; Freiwald and Tsao, 2010) and object-specific representations (cf. Figure 4) in the temporal lobe (Desimone et al., 1984; Haxby et al., 2001; Quiroga et al., 2005) are captured by the simulation results. While there is ample evidence for a hierarchy of timescales in the visual processing streams of humans (Hasson et al., 2008), primates (Murray et al., 2014) and rodents (Piasini et al., 2021), with larger temporal stability in higher areas, the compatibility with deep predictive coding is debated (Piasini et al., 2021). Our simulation results of increasingly large timescales further up in the network hierarchy may help to reconcile predictive coding with the experimental evidence. Coincidentally, this was also found to be true in a recently developed predictive coding model, albeit with only two layers and without explicit error representations (Jiang and Rao, 2022). Compared to emergence of temporal hierarchies purely as a result of dynamics in spiking neurons (van Meegen and van Albada, 2021) or large-scale models (Chaudhuri et al., 2015; Mejias and Wang, 2022), our model provides a complementary account, postulating development of the temporal hierarchy as a consequence of a functional computation: learning invariance by local error minimization.

What novel insights can be extracted about the brain’s putative use of predictive algorithms? Theories of predictive coding range from limiting it to a few functions [such as subtraction of corollary discharges to compensate for self-motion (Leinweber et al., 2017)] and input reconstruction (Rao and Ballard, 1999) to claiming extended versions of it as the most important organizational principle of the brain (Friston, 2010), namely the free energy principle. PC models provide a critical step to make theories of perception and imagery quantitative and falsifiable as well as to guide experimental research (Pennartz et al., 2019). Based on the simulation results, error neurons in higher visual areas operate on a much shorter activity timescale than their representational counterparts. This comparison of distinct subpopulations may provide an additional angle to measuring neural correlates of prediction errors [for a review see (Walsh et al., 2020)], as representation neuron responses have been barely considered in experimental work so far. In combination with work on encoding of errors in superficial, and representations in deep cortical layers (Bastos et al., 2012; Keller and Mrsic-Flogel, 2018; Pennartz et al., 2019; Jordan and Keller, 2020), area- and layer-wise recordings of characteristic timescales could lead to a better understanding of cortical microcircuits underlying predictive coding. Layer-wise investigations also show distinct patterns of feedforward and feedback connectivity (Markov et al., 2014) and information processing (Oude Lohuis et al., 2022). Only with knowledge about these microcircuits, models of finer granularity can be constructed.

## 5. Conclusion

Predictive coding is a theory with great explanatory power, but with unclear scope. Here, we go beyond the original scope of pure input-reconstruction and find that predictive coding networks can additionally solve an important computational problem of vision. Our results are in line with experimental data from multiple species, strengthening predictive coding as a fundamental theory of mammalian perception.

## Data availability statement

The datasets presented in this study can be found in online repositories. The names of the repository/repositories and accession number(s) can be found at: https://github.com/matthias-brucklacher/PCInvariance.

## Author contributions

MB implemented the model, conducted the analyses, and wrote the first draft of the manuscript. All authors contributed to the conception and design of the study throughout the project, manuscript revision, read, and approved the submitted version.

## Conflict of interest

The authors declare that the research was conducted in the absence of any commercial or financial relationships that could be construed as a potential conflict of interest.

## Publisher’s note

All claims expressed in this article are solely those of the authors and do not necessarily represent those of their affiliated organizations, or those of the publisher, the editors and the reviewers. Any product that may be evaluated in this article, or claim that may be made by its manufacturer, is not guaranteed or endorsed by the publisher.

## Footnotes

^1^ https://pypi.org/project/sklearn-sfa/
