# Supplementary material for: Local minimization of prediction errors drives learning of invariant object representations in a generative network model of visual perception
Source: Front Comput Neurosci. 2023 Sep 25;17:1207361. doi: 10.3389/fncom.2023.1207361 (PMC10561268; doi:10.3389/fncom.2023.1207361)
Supplement: Supplementary file 1 [file Data_Sheet_1.pdf]

Supplementary Material

1.1 Network architecture and hyperparameters

The networks consist of four fully connected areas of which the topmost (area 3) has no error units and the lowest (area 0) is driven by the input only, in a pixel-wise manner. Thus, the number of neurons in area 0 corresponds to the resolution of the inputs. Table S1 shows the precise number of neurons per area. Table S2 lists the used hyperparameters.

Network area	Number of neurons
Input: Area 0	1156
Area 1	2000
Area 2	500
Area 3	30

Table S1. Network architecture used for the different datasets.

Parameter	Value	Meaning
ϵ_{inf}	0.05	uniform inference rate for areas 1-3
ϵ_{learn}	0.01	learning rate
n_{eps}	20-40	number of training epochs
n_{isteps}	10	inference steps before weight update
n_{freps}	5	inference-learn cycles on each frame
n_{sreps}	10	cycles on each sequence per epoch

Table S2. Network hyperparameters.

1.2 Training

Network training was conducted in nested loops as described in section 2.3. Alg S1 depicts the pseudocode for both training paradigms. The main difference between the continuous and static training paradigms is the additional reset of activity in the beginning of the fifth ‘for’ loop. To compensate for the shorter time between activity resets in the static training paradigm, the number of cycles per frame n_{freps} is increased by a factor of 6.

```
for i ← 1, n_eps do                                          ▷ Epochs
    for j ← 1 , n_seqs do                                    ▷ Number of sequences
        Initialize activity
        for k ← 1, n_sreps do                                ▷ Iterations per sequence
            for l ← 1, n_fps do                              ▷ Frames per sequence
                for m ← 1,n_freps do                         ▷ Iterations per frame
                    (Static training: Reset activity)
                    for n ← 1,n_isteps do                    ▷ Inference steps
                        inference (Eq. 1-3)
                    end for
                    update weights (Eq. 4)
                end for
            end for
        end for
    end for
end for
```

Alg S1. Pseudocode for network training. Nested loops during network training as described in the Methods section. In the static training, activities are reset each time a new input is presented.

1.3 Inference of high-level representations

Convergence of high-level neural activity took considerably longer (2000 inference steps were used) than the duration that was necessary until the total prediction error in the network converged (one to two orders of magnitude less). I.e., after the prediction errors were minimized, further inference improved invariance, but not stimulus reconstruction. Across the network areas, this is plotted for the rotating digits in Figure S1.

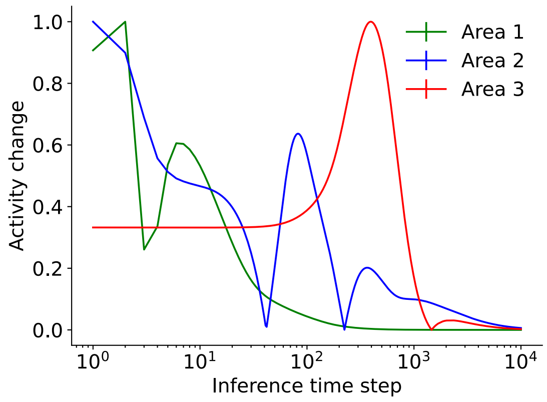

Figure S1. Convergence of activity over inference steps for a fixed inputs from the rotating digits dataset across representation neurons in the different network areas.

1.4 Influence of temporal continuity

In how far is the learning of invariant representations attributable to the training paradigm? Figure S2 shows the RDM of a network trained in a static manner: the more position-dependent representations stand in contrast to the RDMs of the continuously trained networks (Figure 4B-F) with more uniform representations within sequences.

To analyze whether the different neural timescales are a result of the training paradigm or a result of the network architecture, we compared the decay speed of activity autocorrelation to the statically trained network (Figure S3).

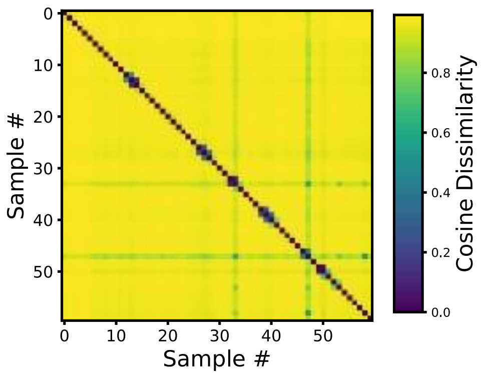

Figure S2. Influence of static training on high-level representational invariance. Network trained in a static manner with resets of activity after each frame show less invariance in area-3 representations compared to the continuously trained networks (Figure 4B-F). The diagonal structure of the RDM shown here reveals that representations are specific to individual images and do not generalize across positions.

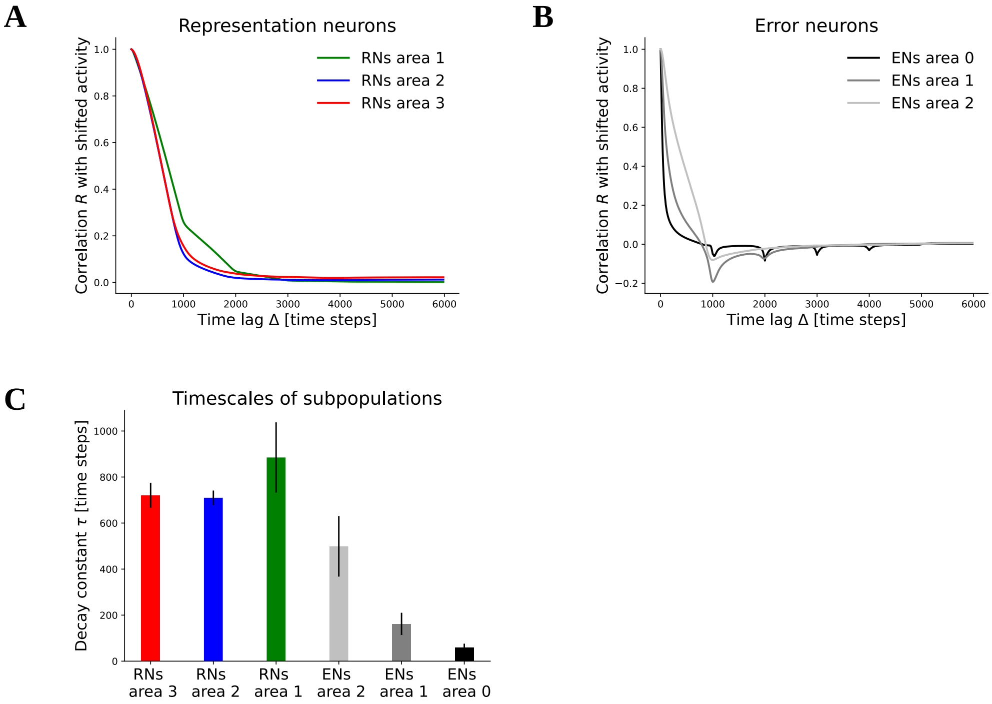

Figure S3. Decay of activity autocorrelation of neural subpopulations in a statically trained network in which activity is reset after each frame. (A) Representation neurons, **(B)** Error neurons, **(C)** Inferred decay constants to quantify the timescale on which neural activity changed. This figure can be compared to Figure 6, but note the different scale in Figure 6C.Reconstruction of complete inputs

1.5 Reconstruction of complete inputs

To test which input patterns can be reconstructed from different network areas, an input image needs to be presented for an extended period. Here, 2000 inference steps were used. The image was then removed, and reconstructions were triggered from the chosen area, i.e., predictions were consecutively projected to the area below according to Figure 2A:

$$\hat{\boldsymbol{y}}_l = \boldsymbol{W}_l \boldsymbol{y}_{l+1} \quad (7)$$

For symbols, the reader is referred to Equation 1.

1.6 Reconstruction of occluded sequences

For reconstruction of whole objects from partially occluded sequences, the first frame of a sequence was presented for an initial number of inference steps (see below) to give higher areas time to infer stimulus identity. Each consecutive frame of the sequence was then shown for a varying number of consecutive inference steps that is specified later. Lastly, reconstructions were normalized, dividing all predictions by the value of the largest pixel-wise prediction of the current reconstruction. To systematically investigate the difference between the static and continuous training paradigm, we varied both the initial and consecutive number of inference steps across a range of parameters and used the optimal combination we found for Figure 8. The continuously trained network consistently achieved better reconstructions than the statically trained network as shown by the consistently positive mean difference in reconstruction errors in Figure S4.

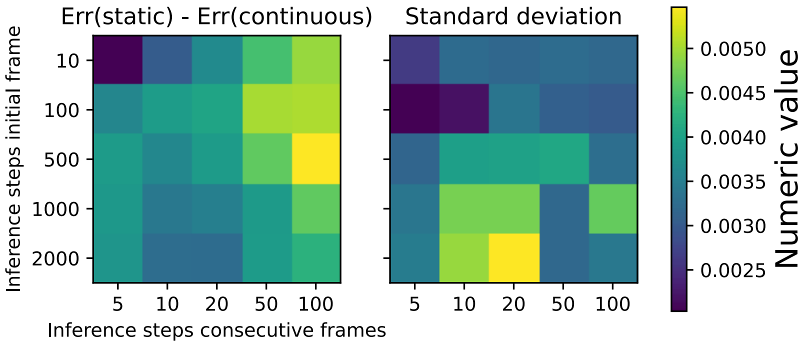

Figure S4. Reconstruction of occluded scenes: influence of hyperparameters. The continuously trained network achieved better reconstructions of occluded inputs than the statically trained one. The left plot shows the mean difference in reconstruction error between the two training paradigms across ten occlusion sequences, for various hyperparameters (see main text). Positive values indicate better performance of the continuously trained network. The right plot shows the standard deviations across the ten occlusion sequences.

1.7 Influence of weight initialization

Initializing weights to lower values than used by default (for default values see section 2.2) led to a decrease in performance, with less distinguishable representations in area 3 after 10 epochs (Figure S5). To test this, we decreased the standard deviation of the Gaussian weight initialization. As the distribution is centered at zero and clipped at zero to prevent negative weights, the value of the standard deviation corresponds to the average value of the initial weights (note that this is before dividing each weight by the number of neurons in the next higher area). Despite initially less distinguishable representations for smaller initial weights, prolonged training led to a non-collapsed state even for the smallest initialization that we tested (on the right of Figure S5).

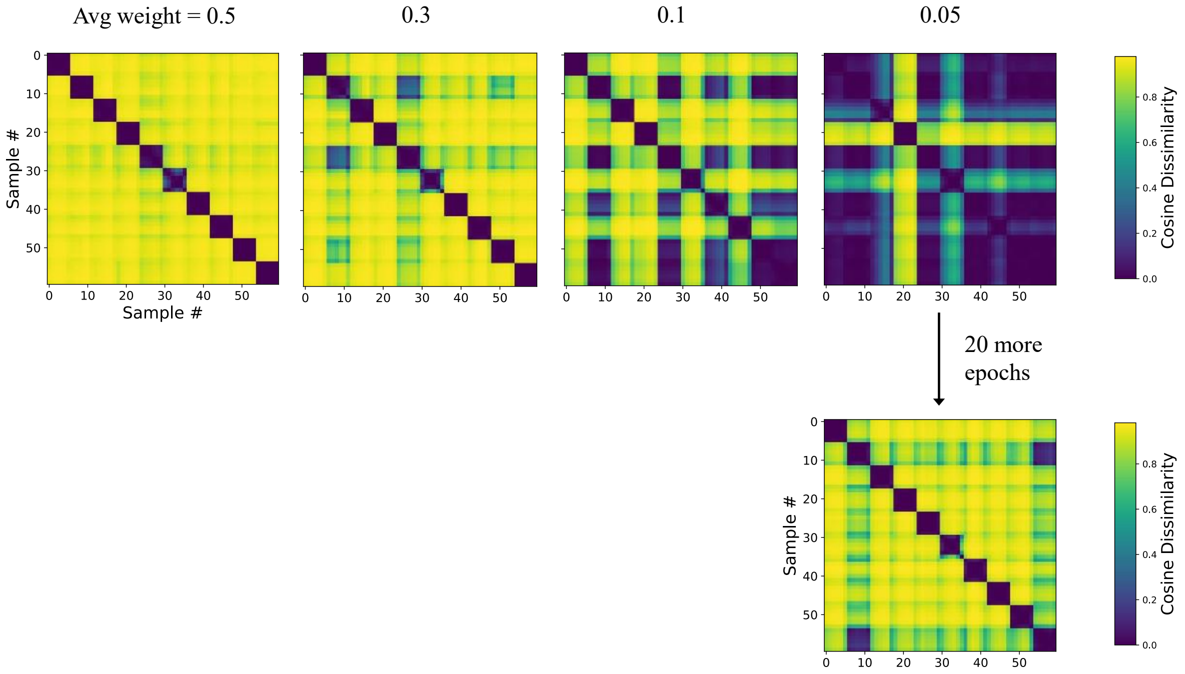

Figure S5. Influence of weight initialization. Initializing weights to smaller values gradually deteriorates the RDMs that are shown after 10 training epochs. The leftmost plot corresponds to the default settings used in the rest of the paper, shown for a digit translation dataset. The rightmost plot corresponds to a tenth of these weight values. After 20 more epochs on the network trained with an initial weight of 0.05, a considerable improvement was observed.

1.8 Representational dissimilarity matrices on larger datasets

To test the influence of increasing the dataset size on the RDMs, we ran the same analysis of constructing the RDMs (as in Figure 4) for the networks trained on the larger datasets. While the RDMs shown in Figure S6 lost the clear block-diagonal structure, decoding performance was still far above chance (Figure 5C). This effect, that was present for both the default and the increased network size,

can be explained by a better approximation of the underlying data-generating distribution when dataset size was increased.

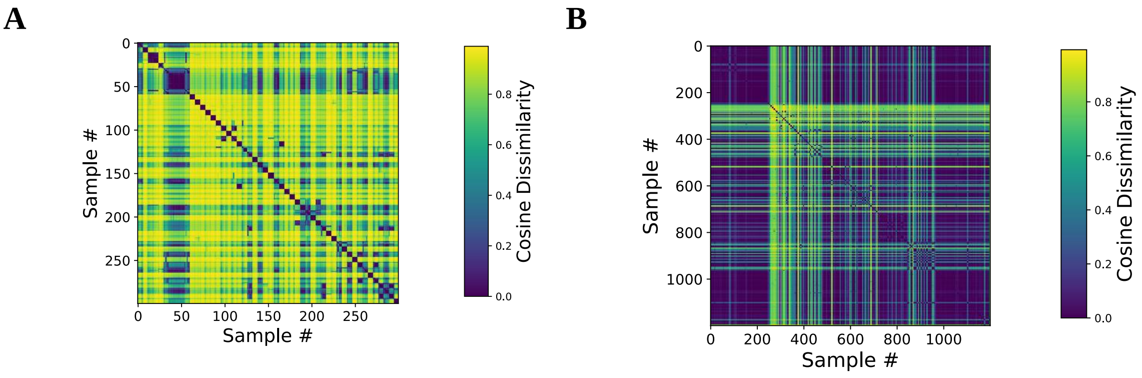

Figure S6. Representational dissimilarity matrices on larger datasets. When dataset size is increased such that multiple digits of each class are shown (in different sequences), the network forms instance-specific (e.g. a particular digit '1' irrespective of position) instead of class-specific (e.g. all digit '1's) representations. **(A)** RDM for a network of increased size, with [4000, 2000, 90] neurons in [area 1, area 2, area 3] (chosen because it better visualizes the effect) instead of the default [2000, 500, 30]. The network was trained and evaluated on a dataset of 50 digit instances (sequences), five '1's, five '2's, etc., resulting in 300 image frames. The blocks along the diagonal are of size six, as each sequence contains six frames. **(B)** When increasing the dataset size even further, the structure in the RDM becomes less obvious, but the digit classes are still relatively well decodable (Figure 5C). An explanation is given in the main text.

1.9 Learning multiple transformations in a single network

Learning different transformations of the same digit when these are not shown in sequence proved challenging. Nevertheless, Figure S7 provides a proof of principle result showing decoding of digit identity with ~80% accuracy after seven training epochs, with a linear decoder again trained on 2/3 of the inferred representations. With prolonged training, the network tended to separate the invariant representations for the different transformations of each digit, suggesting the need for a unifying mechanism to merge these different invariant representations.

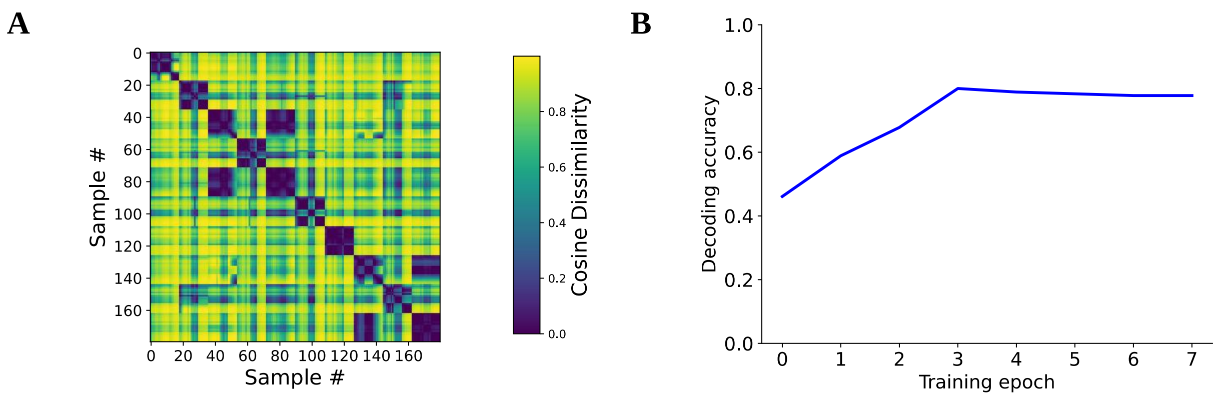

Figure S7. Learning multiple transformations in a single network with [2000,500,10] neurons in [area 1, area 2, area 3]. **(A)** RDM of ten digits each undergoing three transformations (translation, rotation, scaling). **(B)** Linear decoding accuracy of digit identity from area 3 of the same network.

1.10 Comparison of continuously trained to the untrained network

Complementing Figure 4, Figure S8 depicts the comparison of the continuously trained against the untrained network for the remaining datasets (listed in the figure caption).

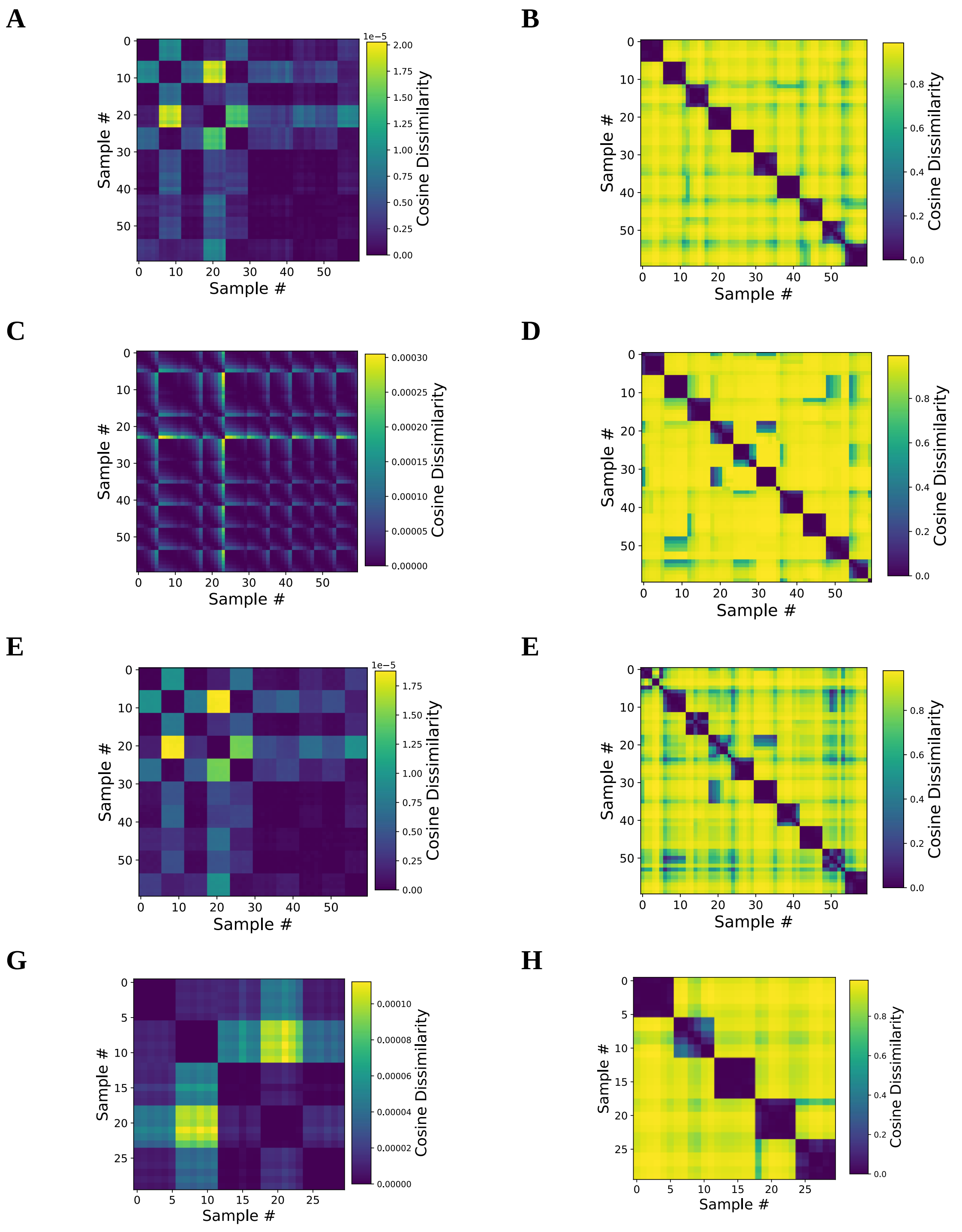

Figure S8. Comparison of continuously trained to the untrained network. Complement to Figure 4: comparison of untrained RDMs (left) to trained (right). **(A)** and **(B)** digit rotation, **(C)** and **(D)** digit scaling, **(E)** and **(F)** digit translation with noise, **(G)** and **(H)** rotating toy objects.

1.11 Comparison across areas

Higher network areas contained more invariant representations than lower areas. We quantified this by computing a sequence invariance value S, that is given as the ratio between the average cosine distance $\bar{d}_{in}$ between representations of frames from the same sequence (after 2000 inference steps each) and the average distance to frames from all other sequences $\bar{d}_{across}$:

$$S = \frac{\bar{d}_{in}}{\bar{d}_{across}}$$

As shown in Figure S9, area 2 initially rises quickest, but is then surpassed by area 3. The result is related to the hierarchy of timescales, as more invariant representations (in areas with a higher value according to Figure S9) can be expected to be more stable across time.

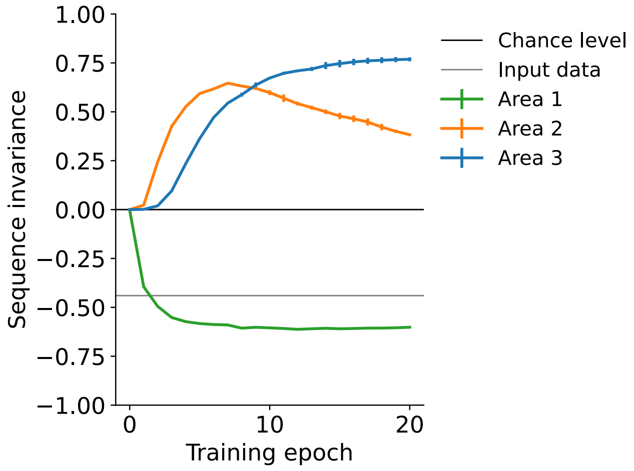

Figure S9. Comparison of representational invariance across network areas. The highest network area (area 3) eventually develops more invariant representations as compared to lower areas, which is mirrored in an eventually larger sequence invariance value. Error bars indicate standard deviation across two random seeds (computed on the fast translation digits).

1.12 Comparison to Slow Feature Analysis

To compare model performance to linear Slow Feature Analysis (SFA) (40), we used the sklearn-sfa package from https://pypi.org/project/sklearn-sfa/. From the concatenated digit/toy object input sequences, we let the algorithm extract a fixed number of features. As this number of features needs to be provided for the SFA algorithm, we tried different settings and optimized for decoding accuracy on each dataset. The subsequent linear decoding analysis consisted of training a linear decoder in a stratified k-fold manner on 2/3 of the extracted features and evaluated on the remaining 1/3. For the ten moving digits, 30 features – matching the number of neurons in our top layer - proved optimal. For the five toy objects, reducing the number of features from 30 to 10 significantly improved accuracy from 23.33% to 73.33%, so we used this setting for the results shown in Fig5a and Tab 1.

1.13 Changing the number of neurons in different network areas

As it could be expected that projecting from the representation in one area to a larger number of neurons in the next higher area orthogonalizes the representations in the higher area, we systematically analyzed the influence of varying the size of all three network areas across almost two orders of magnitude. The results depicted in Figure S10 show the robustness of the learning paradigm and a minimum number of required neurons in area 3. As this analysis was performed on a dataset with ten samples only, we also refer to the simulations on larger datasets (Figure 5c-d) that showed slightly improved performance when network size was increased. Although orthogonalization may play a role, the memorization capacity of area 3 is more important here: the more sequences are shown, the more neurons are necessary to represent them separately.

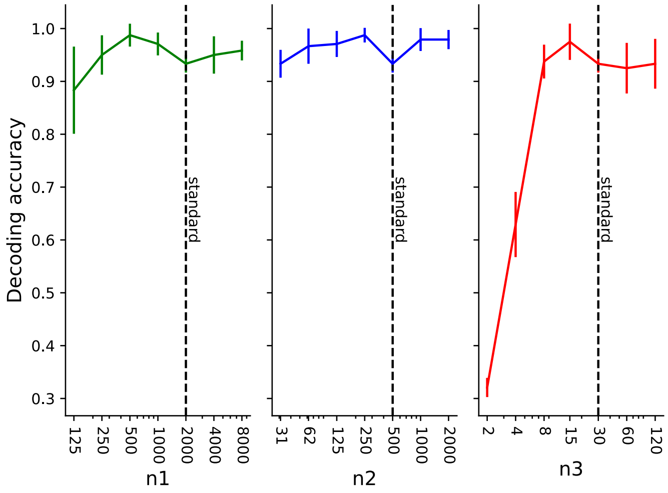

Figure S10. The effect of changing the number of neurons in the respective areas on decoding accuracy of object identity from area 3 representations. On the x-axis, n1 corresponds to the number of neurons in area 1, n2 and n3 accordingly in areas 2 and 3. The vertical "standard" line refers to the network architecture used in most simulations, except where noted differently.

1.14 Influence of sequence order

While the influence of temporal continuity becomes clear when comparing to the static training paradigm (Fig 5b) it is not obvious whether only temporal proximity of input images is necessary or whether the transformation also needs to be spatially continuous. To test this, we trained the network from Fig 5b on shuffled versions of the input sequences. We found that irrespective of sequence order, the only important factor was the temporal continuity of the transformation (Figure S11). In a less ethologically plausible paradigm that would differ significantly from the scope of this paper, this fact could be exploited by showing distinct instances from an object class consequently without resets. In this case, the preselection of which images to show in sequence, however, would require labeled data and thus be supervised. Additionally, it should be noted that movement and spatial continuity are non-trivial concepts in fully connected networks without an a priori retinotopic layout.

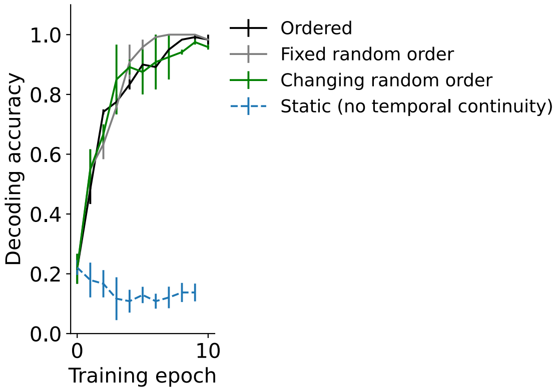

Figure S11. Temporal continuity but not sequence order influences decoding accuracy of object identity. Four conditions are compared: No temporal continuity (the static training paradigm) and three temporally continuous transformations. In addition to the spatially continuous transformation used in the main text ('ordered'), sequences were presented in a fixed random order and in a changing random order that was shuffled newly each epoch.

1.15 Reducing the number of activity resets between sequences

To investigate how representation learning is influenced by not resetting the network activity at the start of each new sequence, we introduced a probabilistic reset. As shown in Figure S12a, resetting the activity approximately every three sequences slightly reduced the linear decodability. This decrease in performance follows from the less separated representational manifolds as shown in the RDMs (Figure 12b). Merging of representations across sequences in turn is caused by distinct digits being now presented in sequence with high probability (a '5' has a 70% chance of being followed by a '6' without activity reset). As mentioned in the main text, such a situation is rare in nature where objects are typically encountered in a randomly interleaved manner, and resets can be provided by head/eye movements, object-based attention and neural replay mechanisms.

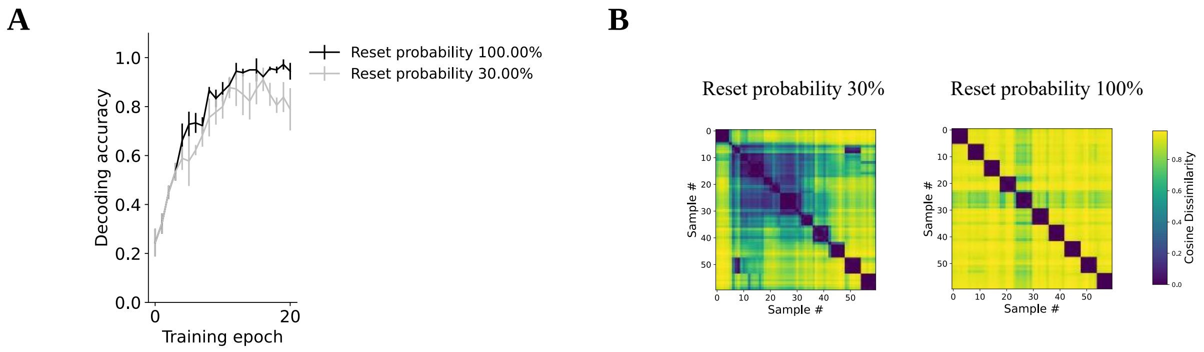

Figure S12. Reducing the number of activity resets maintains well-behaved representation learning. (A) Linear decoding accuracy in the standard training paradigm and with reduced probability of activity reset, shown for the fast digit rotation dataset. Small differences between the RDM shown here to Figure 5B result from sequential instead of batchwise training being deployed here. **(B)** RDM for both conditions from **(A)**.

1.16 Statistical methods to analyze the autocorrelation decay constant

To analyze whether significant differences between dynamics across network areas were present, we first conducted a Welch ANOVA using the *pingouin* package in Python (https://pingouin-stats.org/build/html/index.html). The null hypothesis (i.e., no significant difference in τ-value across error neurons and representation neurons in the three network areas) was rejected based on the p-value of 1.64e-7 (Table S3).

Welch ANOVA

	Source	ddof1	ddof2	F	p-unc	np2
0	Population	5	7.815788	146.460069	1.640665e-07	0.960682

Table S3. Full report on the outcome of the Welch's ANOVA for the intrinsic timescale of neural populations. ddof1: degrees of freedom (numerator), ddof2: degrees of freedom (denominator), p-unc: uncorrected p-values, np2: Partial eta-square effect sizes. See also main text section 3.2 – Temporal stability of representations.

Subsequently, multiple pairwise comparisons were conducted using the *pingouin* implementation of the Games-Howell post-hoc test, which led to the corrected p-values reported in section 3.2 of the main text. All comparisons are shown in Table S4.

Games-Howell post-hoc

	A	B	mean(A)	mean(B)	diff	se	T	df	pval	hedges
0	e0	e1	617.500000	745.000000	-127.500000	33.880919	-3.763180	4.380693	0.093202	-2.313887
1	e0	e2	617.500000	1352.500000	-735.000000	50.621142	-14.519625	5.564737	0.000095	-8.927761
2	e0	y1	617.500000	1347.500000	-730.000000	51.760667	-14.103373	5.470723	0.000125	-8.671818
3	e0	y2	617.500000	6343.476848	-5725.976848	248.192009	-23.070754	3.091242	0.000725	-14.185641
4	e0	y3	617.500000	8724.275103	-8106.775103	867.998896	-9.339615	3.007359	0.012329	-5.742700
5	e1	e2	745.000000	1352.500000	-607.500000	43.180821	-14.068746	3.808161	0.001169	-8.650527
6	e1	y1	745.000000	1347.500000	-602.500000	44.511235	-13.535909	3.756270	0.001449	-8.322898
7	e1	y2	745.000000	6343.476848	-5598.476848	246.782003	-22.685920	3.022249	0.000873	-13.949016
8	e1	y3	745.000000	8724.275103	-7979.275103	867.596777	-9.196986	3.001794	0.012967	-5.655001
9	e2	y1	1352.500000	1347.500000	5.000000	58.273779	0.085802	5.992926	0.999999	0.052757
10	e2	y2	1352.500000	6343.476848	-4990.976848	249.631609	-19.993369	3.162012	0.000988	-12.293432
11	e2	y3	1352.500000	8724.275103	-7371.775103	868.411625	-8.488803	3.013073	0.016134	-5.219557
12	y1	y2	1347.500000	6343.476848	-4995.976848	249.865177	-19.994690	3.173522	0.000968	-12.294244
13	y1	y3	1347.500000	8724.275103	-7376.775103	868.478795	-8.493904	3.014003	0.016091	-5.222693
14	y2	y3	6343.476848	8724.275103	-2380.798255	901.762454	-2.640161	3.480674	0.285854	-1.623370

Table S4. Full report of the post-hoc pairwise test (Games-Howell). Columns A and B refer to the neuronal populations tested in a pairwise manner. Columns: mean: average decay constant across the randomly initialized runs, diff: difference between mean values, se: standard error, df: adjusted degrees of freedom, pval: Games-Howell corrected p-values, hedges: Hedges effect size.
